# Toward Personalized Medicine: The Effect of Treatment of Chronic Enterovirus Diarrhea in an Immunocompromised Patient and the Correlation With In Vitro Models

**DOI:** 10.1093/ofid/ofaf212

**Published:** 2025-04-10

**Authors:** Giulia Moreni, Carlemi Calitz, Gerrit Koen, Hetty van Eijk, Nina Johannesson, Jamy De Ruijter, Kimberley S M Benschop, Jeroen Cremer, Dasja Pajkrt, Adithya Sridhar, Edgar J Peters, Katja C Wolthers

**Affiliations:** Department of Medical Microbiology, OrganoVIR Labs, Amsterdam UMC, Academic Medical Center, University of Amsterdam, Amsterdam Institute for Infection and Immunity, Amsterdam, The Netherlands; Department of Pediatric Infectious Diseases, OrganoVIR Labs, Amsterdam UMC, Academic Medical Center, University of Amsterdam, Amsterdam Institute for Infection and Immunity, Emma Children’s Hospital, Amsterdam, The Netherlands; Department of Medical Microbiology, OrganoVIR Labs, Amsterdam UMC, Academic Medical Center, University of Amsterdam, Amsterdam Institute for Infection and Immunity, Amsterdam, The Netherlands; Department of Pediatric Infectious Diseases, OrganoVIR Labs, Amsterdam UMC, Academic Medical Center, University of Amsterdam, Amsterdam Institute for Infection and Immunity, Emma Children’s Hospital, Amsterdam, The Netherlands; Department of Medical Microbiology, OrganoVIR Labs, Amsterdam UMC, Academic Medical Center, University of Amsterdam, Amsterdam Institute for Infection and Immunity, Amsterdam, The Netherlands; Department of Medical Microbiology, OrganoVIR Labs, Amsterdam UMC, Academic Medical Center, University of Amsterdam, Amsterdam Institute for Infection and Immunity, Amsterdam, The Netherlands; Department of Medical Microbiology, OrganoVIR Labs, Amsterdam UMC, Academic Medical Center, University of Amsterdam, Amsterdam Institute for Infection and Immunity, Amsterdam, The Netherlands; Department of Pediatric Infectious Diseases, OrganoVIR Labs, Amsterdam UMC, Academic Medical Center, University of Amsterdam, Amsterdam Institute for Infection and Immunity, Emma Children’s Hospital, Amsterdam, The Netherlands; Department of Anesthesiology, Amsterdam UMC, Vrije Universiteit Amsterdam, Amsterdam, The Netherlands; National Institute for Public Health and the Environment, Bilthoven, The Netherlands; National Institute for Public Health and the Environment, Bilthoven, The Netherlands; Department of Pediatric Infectious Diseases, OrganoVIR Labs, Amsterdam UMC, Academic Medical Center, University of Amsterdam, Amsterdam Institute for Infection and Immunity, Emma Children’s Hospital, Amsterdam, The Netherlands; Department of Medical Microbiology, OrganoVIR Labs, Amsterdam UMC, Academic Medical Center, University of Amsterdam, Amsterdam Institute for Infection and Immunity, Amsterdam, The Netherlands; Department of Pediatric Infectious Diseases, OrganoVIR Labs, Amsterdam UMC, Academic Medical Center, University of Amsterdam, Amsterdam Institute for Infection and Immunity, Emma Children’s Hospital, Amsterdam, The Netherlands; Emma Center for Personalized Medicine, Amsterdam UMC, Amsterdam, The Netherlands; Division of Infectious Diseases, Department of Internal Medicine, Amsterdam UMC, Vrije Universiteit Amsterdam, Amsterdam, The Netherlands; Infectious Diseases, Amsterdam Institute for Infection and Immunity, Amsterdam, The Netherlands; Department of Medical Microbiology, OrganoVIR Labs, Amsterdam UMC, Academic Medical Center, University of Amsterdam, Amsterdam Institute for Infection and Immunity, Amsterdam, The Netherlands

**Keywords:** chronic diarrhea, enterovirus C species, immunocompromised patient, organoids, personalized medicine

## Abstract

Enteroviruses (EV) usually cause acute, mild, self-limiting disease. Chronic infections with EVs are rare, and typically occur in patients with immunodeficiency, posing a high risk of severe outcomes. We report a rare case of chronic diarrhea caused by coxsackievirus A1 (CVA1) (from EV-C species) infection in a patient with a common variable immunodeficiency, who was on treatment with pooled intravenous immunoglobulin (IVIG) from the Netherlands. To explore treatment options, we assessed the presence of neutralizing antibodies (nAbs) against CVA1 in pooled IVIG from South Africa, where EV-Cs are prevalent, and tested the antiviral efficacy of US Food and Drug Administration–approved drugs like fluoxetine, itraconazole, ribavirin, and remdesivir (RDV) against CVA1 in vitro. Both Dutch and South African IVIG showed low nAb titers against CVA1. The patient, treated with Dutch IVIG, also received a combination of amantadine and fluoxetine, which were discontinued due to side effects. Among the drugs tested, only RDV significantly inhibited CVA1 replication in rhabdomyosarcoma (RD) cells. This in vitro efficacy was not reflected by a favorable clinical response after treatment of the patient with RDV. In concordance with unfavorable antiviral response in the patient, preliminary tests on a co-culture model containing isogenic human intestinal cells and intestinal fibroblasts showed no significant reduction in CVA1 RNA copies after RDV administration. In conclusion, our results showed that repurposing of drugs that have shown in vitro efficacy does not translate well to the patients, and this is also reflected in a more physiologically relevant model of the human intestine.

Enteroviruses (EVs) are nonenveloped, single-stranded RNA viruses from the Picornaviridae family [1], with species *Enterovirus alphacoxsackie* (EV-A), *Enterovirus betacoxsackie* (EV-B), *Enterovirus coxsackiepol* (EV-C), and *Enterovirus deconjuncti* (EV-D) important for human infections. Generally, EV infections cause mild respiratory or gastrointestinal symptoms, but patients with X-linked agammaglobulinemia and common variable immunodeficiency (CVID) are particularly susceptible to severe EV infections [2, 3], such as chronic enteroviral meningoencephalitis in agammaglobulinemia. A rare clinical manifestation associated with EV infections is chronic diarrhea [4–6]. In 1 post–kidney transplant case, this was caused by coxsackievirus A19 (CVA19) [7].

We report a 28-year-old adult CVID patient with severe chronic diarrhea diagnosed with coxsackievirus A1 (CVA1) infection. CVA1 belongs to EV-C as the abovementioned CVA19 and can cause diarrhea [8], which can become persistent and life-threatening in immunosuppressed patients [9]. The patient likely contracted CVA1 during a trip to Africa, as EV-C is sporadically detected in Europe [10]. While intravenous immunoglobulins (IVIGs) can protect immunocompromised patients from circulating viruses [11], they offer no protection against rarely circulating genotypes like CVA1.

There are no effective antivirals against EV registered [12], and effective antivirals against EV are urgently needed [13]. Although novel compounds have shown efficacy against EV infections in vitro, due to safety concerns the United States Food and Drug Administration (FDA) did not approve their use in humans [14, 15]. Nevertheless, several drugs that are European Medicines Agency or FDA approved for other indications have shown antiviral activity against EV infection in vitro. The antidepressant fluoxetine (FLX) has shown efficacy against coxsackievirus B3 and EV-D68 [16], while itraconazole (ITZ) has been effective against echovirus 30 [17], EV-A71 [18], chikungunya virus [19], and severe acute respiratory syndrome coronavirus 2 (SARS-CoV-2) [20]. Ribavirin (RBV), a guanosine nucleoside analogue, was shown to be effective against EV-A71 infection on rhabdomyosarcoma (RD) cells and in mice [21], and remdesivir (RDV), an adenosine nucleoside analogue, showed inhibition of hepatitis C virus, Ebola virus, and SARS-CoV-2 [22]. In this study, we evaluated the toxicity and efficacy of 4 FDA-approved drugs (FLX, ITZ, RBV, and RDV) against CVA1 in cell lines and a human organotypic model of the intestine. We also assessed the neutralizing potential of IVIGs from the Netherlands and South Africa against CVA1.

## MATERIALS AND METHODS

### Virus Detection

EV detection from stool samples collected from a patient admitted to hospital was performed by standard real-time polymerase chain reaction (PCR) and genotyping by sequencing the VP1 region, revealing CVA1 [23]. The fecal material was also passaged 3 times on HT29 cells (HTB-38 ATCC), RD cells (CCL-136 ATCC), and Vero cells (kindly provided by the National Institute of Public Health and the Environment, the Netherlands), which were cultured as previously described [24]. Cytopathogenic effect was observed in RD cells, as previously reported [25]. The viral genotype was confirmed by sequencing after isolation on RD cells.

### Next-Generation Sequencing

A clinical stool sample positive for CVA1 was collected from the patient and sequenced following a previously published procedure [26]. The PCR assay used is based on a previously published article [27]. The sequencing was performed using minION with Ligation Sequencing Kit 14 with Native Barcoding kit 96 and Flow Cell 10.4.1. Near full-length fastq data were analyzed with Canu V2.1 parameter options: minReadLength = 6100; minOverlapLength = 6000; corOutCoverage = 1500; correctedErrorRate = 0.005; canuIterationMax = 10; maxInputCoverage = 8000. The contigs of the sample are compared with Bionumerics version 7.6 maximum parsimony trees. The full-length nucleotide sequence of the CVA1 patient isolate pretreatment is available in GenBank nucleotide sequence databases (accession number PQ303586). The full-length sequence of the CVA1 patient isolate posttreatment is available on Figshare.

### Cells and Organoid Cultures

#### Human Fetal Intestinal Enteroids and Primary Human Intestinal Fibroblasts

Enteroids were obtained by isolating crypts from fetal intestinal tissue (gestational age, 14–16 weeks, sex unknown) as previously described [28]. Crypts were embedded in Matrigel (Corning, New York, New York, USA), seeded according to Roodsant et al [29], and maintained in complete IntestiCult Organoid Growth Medium (Stemcell Technologies, Cambridge, United Kingdom). The remaining tissue was digested in Collagenase A (Sigma-Aldrich, St Louis, Missouri, USA) for 45 minutes at 37°C, after which collagenase activity was inhibited using 0.05 mM ethylenediaminetetraacetic acid. The resulting cell suspension was passed through a 70-µm cell strainer, centrifuged at 300*g* for 5 minutes, and resuspended in Advanced Dulbecco’s modified Eagle medium (AdvDMEM)/F12 supplemented with 1% (v/v) Pen-Strep, 1% (v/v) Glutamax, and 2% (v/v) fetal bovine serum (FBS) (2% AdvDMEM/F12). Primary human intestinal fibroblasts were allowed to adhere to the culture flask overnight and medium was replenished thereafter. Intestinal fibroblasts (IFs) were routinely cultured at 37°C, 5% carbon dioxide (CO_2_), and 95% humidity in 2% AdvDMEM/F12, with medium change every other day. Cells were subcultured by trypsinization upon reaching 70% confluence.

#### Human Intestinal Epithelial Cultures

Human intestinal epithelial (HIE) cultures were obtained as previously described [30]. In brief, HIE cultures were established from human fetal enteroids dissociated into single cells, seeded onto laminin-coated inserts in differentiation medium with supplements, and maintained with regular medium changes. By day 14, monolayers with trans-epithelial electrical resistance (TEER) >200 Ω·cm² were used for infection.

#### Human Fetal Intestinal Fibroblasts and HIE Isogenic Co-culture

Co-cultures were obtained as previously published [31]. Co-cultures of HIE with IFs were maintained in 300 µL and 700 µL of IntestiCult Organoid Differentiation Medium human (ODMh) medium in the apical and basolateral compartment, respectively, and culture conditions were the same as the HIE cultures. The term “isogenic co-cultures” describes cultures formed by seeding primary human IFs alongside HIE isolated from the same human donor.

### Virus Infection and Antiviral Testing

CVA1 clinical isolate was diluted to 10^2.3^ tissue culture infectious dose 50 (TCID_50_)/mL in Eagle's Minimum Essential Medium (EMEM) with 2% FBS (2% EMEM) for infection on RD cells and 10^4^ TCID_50_/mL in ODMh medium for HIE or HIE co-cultures. A volume of 100 µL was added per well, basolaterally for HIE. RD cells were incubated for 1.5 hours at 37°C, while HIE cultures were incubated for 2 hours. Following incubation, RD cells were washed 3 times with 2% EMEM while HIE cultures were washed with AdvDMEM, then incubated for 10 minutes at 37°C before medium collection. Samples (200 µL/well, both apically and basolaterally for HIE) were collected at 0, 24, 48, and 72 hours postinfection (hpi) for RD cells and at 0, 72, and 120 hpi for HIE cultures. Antivirals were diluted in 2% EMEM or ODMh medium to a concentration of 1, 1.5, 2, and 2.5 µM ITZ (Sigma-Aldrich); 1.25, 2.5, 5, and 10 µM FLX (Sigma-Aldrich); 10, 20, 40, and 80 µM RBV (AG Scientific, San Diego, California, USA); 4, 8, 16, and 32 µM RDV, also known as GS-5734 (Cayman Chemical, Ann Arbor, Michigan, USA); and 3.3 µM of amantadine (AMD) (MedChemExpress, Monmouth Junction, New Jersey, USA). A volume of 200 µL/well of medium alone or with antivirals was added and cells were incubated at 37°C. Antivirals were administered following sample collection. Antiviral dosages were based on the maximum plasma concentration (C_max_) observed in humans as previously described [32]. Viral copies were determined by quantitative reverse-transcription PCR (RT-qPCR) [24].

### Adenosine Triphosphate Assay

To determine cell viability upon treatment with different concentrations of antivirals, intracellular adenosine triphosphate (ATP) was measured at 72 hpi using CellTiter-Glo 3D Cell Viability Assay (Promega, Madison, Wisconsin, USA) following Wrzesinski et al [33] with minor modifications for transwell cultures. A standard curve for ATP disodium salt hydrate (Sigma-Aldrich) was used to normalize data.

### Neutralization Assay

Neutralizing antibody (nAb) titers of 2 Dutch IVIG batches (Nanogam, Sanquin, the Netherlands) manufactured in 2019 and in 2021, and 1 South African IVIG batch (Polygam, National Bioproducts Institute, Pinetown, South Africa), against CVA1 and herpes simplex virus type 1 (HSV-1) were determined. HSV-1 served as positive control due to its high prevalence in both Africa and Europe [34]. Two-fold serial dilutions of IVIG were incubated in duplicates with an equal volume (50 µL) of 100 TCID_50_/mL suspension of viral stock for 1 hour at 37°C, 5% CO_2_. After incubation, 100 µL of RD cells for CVA1 and 100 µL of HEL cells for HSV-1 were added and incubation was carried out at 37°C, 5% CO_2_. After 4 days, nAb titers were calculated based on cytopathogenic effect using the Reed-Muench method [35]. Titers >1:16 were considered seropositive [11]. Titers between 1:16 and 1:32 were considered low and not associated with protection [36].

### Statistical Analysis

Experiments were performed in triplicate in 2 biological replicates and the data are presented as mean ± standard error of the mean. Statistical analysis were performed using GraphPad Prism 8 software (GraphPad Software, San Diego, California, USA). A 2-way analysis of variance test with Dunnett multiple comparison was used to determine the statistical significance of quantitative PCR data comparing treated conditions with untreated infected controls and the 0 days postinfection time point with later time points and ATP data comparing treated conditions and untreated mock control. *P* values <.05 were considered statistically significant.

### Ethical Approval

The patient gave her written consent to present her case.

### Ethics Statement

Human fetal intestinal tissue, gestational age 14–16 weeks, was obtained from a private clinic by the HIS Mouse Facility of the Amsterdam University Medical Center (UMC). All donors supplied written informed consents for the use of fetal material for research purposes. These consent forms are kept at the clinic and the information available to the Amsterdam UMC does not allow identification of the donor without disproportionate efforts. The use of the anonymized material for medical research purposes is covered by Dutch law (Wet foetaal weefsel and Article 467 of Behandelingsovereenkomst).

## RESULTS

### Clinical Case

A 28-year-old Dutch woman with a history of idiopathic thrombocytopenic purpura since 11 years of age was evaluated for chronic diarrhea. Posttreatment at 13 years of age, she developed persistent hypogammaglobulinemia with low levels of immunoglobulin A (IgA; 0.16 g/L), immunoglobulin M (<0.04 g/L), and immunoglobulin G (IgG; 3.6 g/L) and recurrent upper respiratory infections, leading to a diagnosis of CVID. She received regular IVIG (50 g every 3 weeks) as well as trimethoprim-sulfamethoxazole prophylaxis. At age 23 years, during a trip to South Africa and Tanzania, her IVIG was suspended but prophylaxis continued. Upon returning, she had diarrhea, respiratory infections, and a positive purified protein derivative (PPD) test (negative interferon-γ release assay), with no chest X-ray abnormalities. She later developed mild bronchiectasis, and IVIG therapy was resumed, stabilizing IgG levels at 9–10 g/L. At age 26 years, genetic testing (446 genes) showed no mutations for primary immunodeficiency. Colonoscopy revealed unspecified colonic inflammation, and tests for *Giardia lamblia*, bacteria, and viruses were negative except for persistent CVA1 infection. Despite treatment with amantadine and fluoxetine, based on a previous report by Benschop et al [14], the patient experienced general malaise and discontinued therapy after 10 days. Her stool samples remained positive for CVA1, with no neurological symptoms and a stool frequency of approximately 6 times a day.

### In Vitro Susceptibility of CVA1 to Dutch and South African IVIG

**Figure 1.**
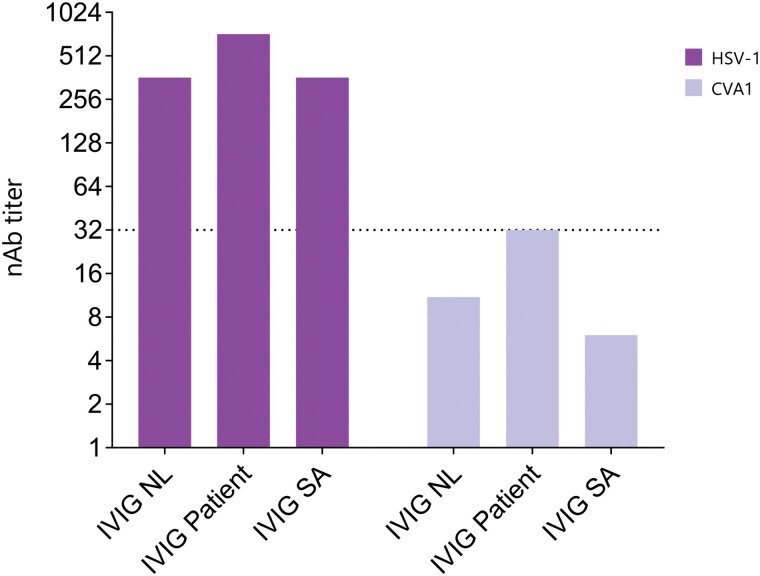
Neutralizing antibody titers in different batches of intravenous immunoglobulin (IVIG) from the Netherlands and South Africa against herpes simplex virus type 1 and coxsackievirus A1. The dotted line indicates the threshold above which the IVIG is neutralizing [28]. Abbreviations: CVA1, coxsackievirus A1; HSV-1, herpes simplex virus type 1; IVIG NL, Dutch batch of intravenous immunoglobulin from 2019; IVIG patient, Dutch batch of intravenous immunoglobulin from 2021 used as substitution therapy in the patient; IVIG SA, South African intravenous immunoglobulin batch; nAb, neutralizing antibody.

As the patient was infected with an EV-C genotype that has not been found to circulate within the Netherlands [37] and given the timing of infection, the chronic intestinal infection could have been acquired during the patient's trip to South Africa and Tanzania. As we hypothesized that the patient might benefit from IVIG supplement containing high levels of type-specific nAb, we tested IVIG that was pooled in South Africa as well as IVIG pooled in the Netherlands. Both IVIG batches showed moderate to high nAb titers against our positive control, HSV-1, which is widely prevalent in both countries. However, the 2 IVIG batches demonstrated very low nAb titers (<32) against CVA1 isolated from the patient (Figure 1).

### Repurposing Clinically Approved Compounds

**Figure 2.**
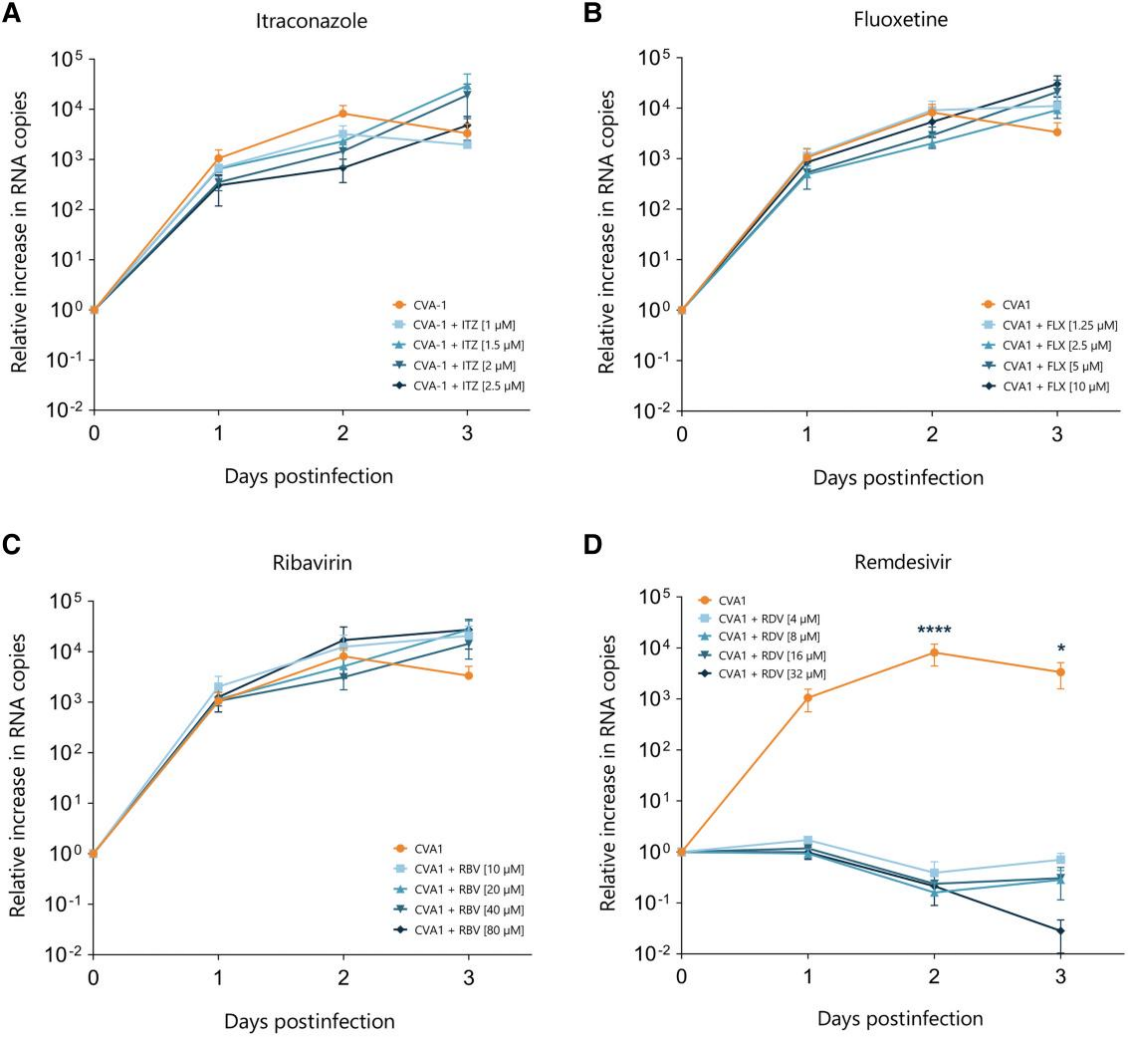
Effect of clinically approved compounds on viral replication of coxsackievirus A1 (CVA1) in rhabdomyosarcoma (RD) cells. *A*, Relative increase in viral RNA copies upon treatment with itraconazole at 1 µM, 1.5 µM, 2 µM, and 2.5 µM. *B*, Relative increase in viral RNA copies upon treatment with fluoxetine at 1.25 µM, 1.5 µM, 5 µM, and 10 µM. *C*, Relative increase in viral RNA copies upon treatment with ribavirin at 10 µM, 20 µM, 40 µM, and 80 µM. *D*, Relative increase in viral RNA copies upon treatment with remdesivir at 4 µM, 8 µM, 16 µM, and 32 µM. The data represent mean ± standard error of the mean of 3 technical replicates in 2 biological replicates. *P < .05, ****P < .0001. Abbreviations: CVA1, coxsackievirus A1; FLX, fluoxetine; ITZ, itraconazole; RBV, ribavirin; RDV, remdesivir.

Repurposing clinically approved drugs can be a useful strategy when no other treatments are available [38]. To test the efficacy of drugs approved for other indications (ITZ, FLX, RBV, and RDV) against CVA1, we first isolated the virus by culturing the original stool sample from the patient on RD cells. Sequencing analysis revealed no significant amino acid changes between the original fecal material and the virus passaged on RD cells (data not shown). RD cells were then infected with CVA1 and treated daily for 72 hours with 1 of ITZ, FLX, RBV, or RDV. We observed that ITZ, FLX, and RBV did not reduce the number of CVA1 RNA copies at any of the concentrations tested (Figure 2*A–C*). RDV completely inhibited CVA1 replication at all 4 concentrations used (Figure 2*D*). Finally, we assessed cell viability of RD cells upon treatment with the different concentrations of all 4 drugs by measuring the release of intracellular ATP at 72 hpi. RD cells treated with ITZ, FLX, and RBV showed no toxicity with similar viability compared to the untreated control (data not shown).

### Patient's Response to Treatment With RDV

**Figure 3.**
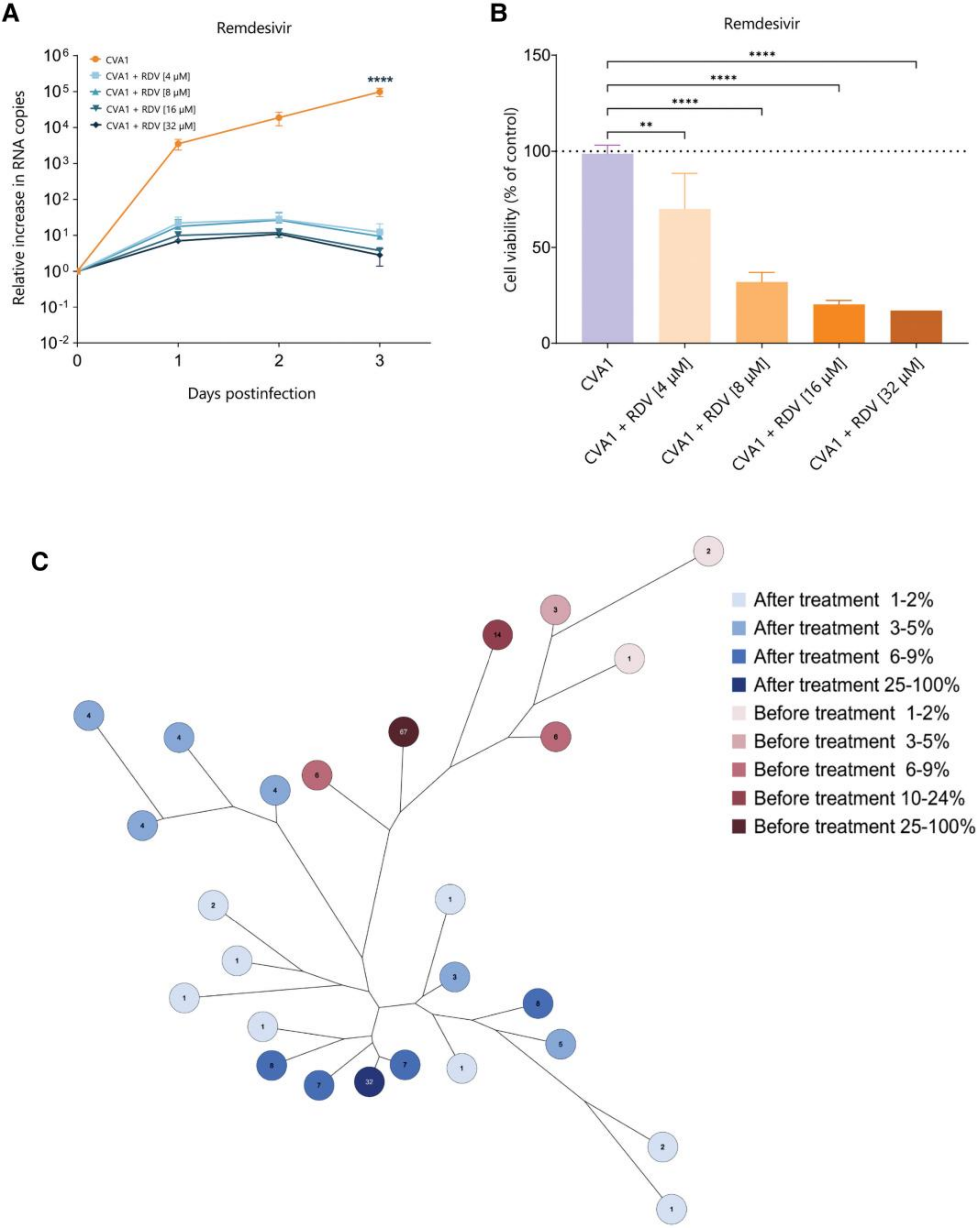
Effect of remdesivir (RDV) on viral replication and quasi-species composition of coxsackievirus A1 (CVA1) in rhabdomyosarcoma (RD) cells. *A*, Relative increase in viral RNA copies upon treatment with RDV at 4 µM, 8 µM, 16 µM, and 32 µM. *B*, Intracellular adenosine triphosphate release upon treatment with RDV relative to untreated control indicated as percentage in RD cells at 72 hours postinfection. The data represent mean ± standard error of the mean of 3 technical replicates in 2 biological replicates. The orange line indicates CVA1 replication on RD cells without any treatment; the blue lines indicate CVA1 replication on RD cells upon treatment with different concentration of antiviral compounds. *C*, Maximum parsimony tree of CVA1 strains isolated from the patient before (red) and after (blue) receiving RDV treatment. The numbers on the tree represent the percentage of sequencing reads that align to each contig, indicating the relative abundance of each contig within the viral population and the coexisting quasi-species before and after treatment. **P < .01, ****P < .0001.

Based on previous in vitro studies demonstrating RDV's efficacy against different EVs [39–41] and a recent case report where a patient with SARS-CoV-2 eliminated vaccine-derived poliovirus after decades of shedding following RDV administration [42], and considering RDV's strong inhibition of CVA1 in RD cells, RDV treatment was initiated. The patient received 100 mg RDV per day intravenously for 10 days with a 200-mg loading dose. Treatment response was assessed by comparing the quantification cycle (Cq) from RT-qPCR on fecal samples collected from the patient before and 10 days after treatment, as well as by monitoring the frequency and severity of diarrheal episodes throughout the treatment period. As there was no increase in Cq levels (indicative of decreasing CVA1 viral load) and diarrhea persisted throughout the treatment period and after its completion, we considered RDV treatment ineffective. To determine if CVA1 had developed resistance to RDV, CVA1 was isolated from a posttreatment fecal sample, showing similar replication reduction as pretreatment isolates (Figure 3*A* and 3*B*). Although resistance to RDV was not observed, the CVA1 isolate post–RDV treatment showed greater genetic divergence compared to the pretreatment isolate, as illustrated in the maximum parsimony tree (Figure 3*C*).

### Antiviral Testing in Organoids

**Figure 4.**
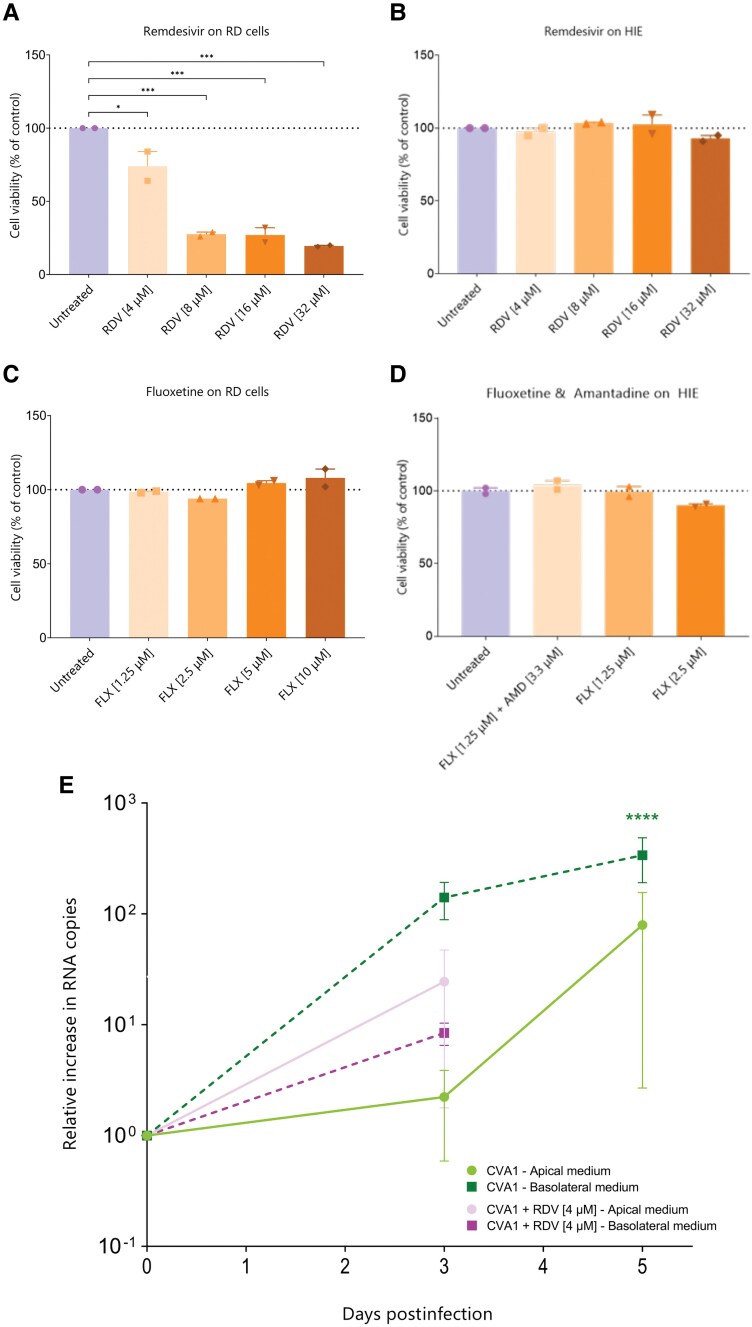
Antiviral testing on organoids. Cell viability at 72 hours after treatment with remdesivir (RDV) and effect of on viral replication of coxsackievirus A1 (CVA1) in a co-culture of human intestinal epithelium (HIE) with fibroblasts. *A*, Intracellular adenosine triphosphate (ATP) release upon treatment with RDV at 4 µM, 8 µM, 16 µM, and 32 µM relative to untreated control indicated as percentage in rhabdomyosarcoma (RD) cells. *B*, Intracellular ATP release, indicated as percentage, upon treatment with RDV at 4 µM relative to untreated control in HIE. *C*, Intracellular ATP release, indicated as percentage, upon treatment with fluoxetine (FLX) at 1.25 µM, 2.5 µM, 5 µM, and 10 µM relative to untreated control in RD cells. *D*, Intracellular ATP release, indicated as percentage, upon treatment with FLX at 1.25 µM together with amantadine at 3.3 µM, or FLX at 1.25 µM, or FLX at 2.5 µM relative to untreated control in HIE. *E*, Effect of RDV on viral replication of CVA1 in a co-culture of HIE with fibroblasts. Relative increase in viral RNA copies upon treatment with RDV at 4 µM from medium collected from both the apical and the basolateral compartment. Statistical significance represents comparisons between time points (0 vs 3 or 5 days postinfection). The data represent mean ± standard error of the mean of 2 biological replicates. For each biological replicate, the data point plotted is the mean of 3 technical replicates. *P < .05, ***P < .001, ****P < .0001. Abbreviations: AMD, amantadine; CVA1, coxsackievirus A1; FLX, fluoxetine; HIE, human intestinal epithelium; RD cells, rhabdomyosarcoma cells; RDV, remdesivir.

As the results in RD cells did not match the clinical outcome, we tested RDV efficacy using a HIE model. However, CVA1 showed a low growth rate on HIE, preventing RDV evaluation (data not shown). Despite this, we assessed the toxicity of RDV and other drugs on HIE compared to RD cells. RDV caused a dose-dependent decrease in viability in RD cells (75% at 4 µM and <25% at higher concentrations; Figure 4*A*), but showed no toxicity in the HIE model (Figure 4*B*). The administration of FLX, which the patient poorly tolerated, showed no toxicity in either model (Figure 4*C* and 4*D*), alone or in combination with AMD. Given the minimal growth of CVA1 on HIE, we established an isogenic co-culture of a HIE with IFs, which was permissive to CVA1 infection as demonstrated by a statistically significant increase in viral copies at 5 dpi compared to 0 dpi. RDV showed only a partial reduction in viral load 72 hpi, which was not significant (Figure 4*E*).

## DISCUSSION

We introduced a patient with CVID and a history of idiopathic thrombocytopenic purpura who developed chronic diarrhea due to a CVA1 infection after traveling to South Africa, where EV-C is prevalent. In addition to the conventional substitution therapy involving IVIG pooled from Dutch donors, treatment with AMD and FLX was initiated and discontinued shortly thereafter due to side effects. In vitro assessments revealed that these drugs lacked antiviral effects in RD cells and were nontoxic. We then tested IVIG from South African donors, which surprisingly showed low nAb titers against CVA1, similar to Dutch IVIG.

We explored the antiviral efficacy of clinically approved drugs (ITZ, FLX, RBV, and RDV), finding that only RDV significantly inhibited CVA1 replication in RD cells. However, the clinical response did not match the in vitro results, as the patient's stool samples remained positive for CVA1 and symptoms persisted. Toxicity tests showed that a clinically relevant concentration of RDV caused 25% cell death in RD cells but was safe in the HIE model. Subsequent tests on an isogenic co-culture of HIE with intestinal fibroblasts revealed a nonsignificant reduction in viral load in the basolateral compartment following RDV treatment. In the apical compartment, however, a slight increase was observed, likely due to greater variability as indicated by the more pronounced error bars compared to the basolateral compartment, as the apical side is not in contact with fibroblasts.

Immunocompromised patients, particularly those with primary immunodeficiencies such as X-linked agammaglobulinemia and CVID, are highly susceptible to EV infections. However, infections caused by EV-C are relatively rare in Europe [43, 44], with only sporadic circulation of EV-C genotypes reported. A recent study conducted in the Netherlands highlighted low nAb levels against EV-C in Dutch IVIG [11]. In contrast, EV-C is more prevalent in Africa [45–47], suggesting that travel to this region increases the risk of exposure to these genotypes. Notably, the patient's symptoms and CVA1 infection developed following a trip to South Africa and Tanzania.

The patient described here experiences persistent, debilitating diarrhea. To explore treatment options, we considered IVIG from South Africa due to the absence of nAbs against EV-C in Dutch IVIG [11]. However, South African IVIG also failed to neutralize CVA1 isolates from the patient, indicating insufficient basis for this treatment. This suggests that CVA1 may be rare among EV-C genotypes in South Africa, consistent with a study showing only 2% prevalence of CVA1 compared to other EV-C genotypes [46]. Additionally, a study in Tanzania found few EV-C genotypes among children with fever and respiratory illnesses, but notably no CVA1, despite these being atypical presentations for EV-C infections [48]. Another potential therapeutic approach could involve using immunoglobulin batches sourced from individuals recently infected with CVA1. While such cases may be infrequent, this strategy might offer an opportunity to obtain antibodies with neutralizing activity specific to CVA1.

Interestingly, several studies indicated the silent circulation of EV-C showing a discrepancy between wastewater and clinical samples in Europe [43, 49, 50], where these viruses are present in the sewage but are absent in clinical samples, a pattern also observed in the United States [51]. These findings suggest higher-than-expected circulation of EV-C in Europe and the United States, but low nAb titers against EV-C in IVIG [11] indicate that EV-C may be less prevalent in these populations than in Africa. Infrequent detection in clinical material may be due to asymptomatic infections or mild symptoms that evade surveillance. We hypothesize that asymptomatic individuals shedding the virus may develop few to no antibodies, as observed in patients with asymptomatic coronavirus disease 2019 [52]. Additionally, poliovirus vaccination may provide population immunity, preventing infections from progressing to symptomatic disease through cross-reactive immunity. The primary role of IgA at mucosal sites, as previously demonstrated for rotavirus [53, 54], also suggests a need to reconsider the exclusive reliance on IgG in IVIG formulations.

Despite CVA1-induced diarrhea, the patient did not develop central nervous system (CNS) complications, which are common in immunocompromised individuals infected with EVs [13]. This may be due to individual diversity, or it might suggest that CVA1 rarely causes CNS symptoms as seen for poliovirus where CNS complications are stochastic [55]. Additionally, the patient's positive PPD suggests that T-cell immunity is at least partially intact, which may have contributed to the absence of CNS involvement in this case.

The lack of nAbs in IVIG led us to explore alternatives by testing in vitro clinically approved compounds known to be active in vitro against EVs. Our results showed that RDV effectively suppressed CVA1 replication in RD cells, although it reduced cell viability by 25% at a clinically relevant concentration of 4 µM, corresponding to the C_max_ values observed in humans, and exhibited high toxicity at higher concentrations.

Based upon published results showing inhibition of EV replication by RDV both in vitro and in a poliovirus-infected patient [39–42], together with the successful inhibition of the CVA1 on RD cells and the experience of using this drug in patients infected with SARS-CoV-2 [56], the off-label treatment with RDV was initiated in the patient for compassionate use. Unexpectedly, the patient did not respond positively to the treatment with RDV. A possible explanation for the treatment failure in this patient is that RDV alone may not be sufficiently effective. However, combining RDV with other antivirals could enhance its efficacy, as suggested by a study demonstrating RDV's synergistic potential when used with rupintrivir [57]. In addition to combination therapies, alternative antiviral candidates such as pocapavir, pleconaril derivatives, and protease inhibitors also show promise due to their ability to inhibit enteroviral replication through distinct mechanisms [58]. These drugs, however, were not tested in this case due to contraindications in patients of childbearing potential. While not feasible for this patient, such agents remain valuable candidates for future research in appropriate populations. Furthermore, evaluating approved RDV analogues together with picornaviral-targeting therapies could provide additional insights into optimizing treatment strategies.

We confirmed that the virus did not develop resistance to RDV, as posttreatment isolates remained susceptible to RDV treatment. However, following RDV administration, we observed a greater variety of quasi-species in the CVA1 sample, suggesting that RDV applied selective pressure on the virus, potentially leading to viral adaptation. While RDV alone did not fully inhibit CVA1 infection, the selective pressure it exerted points to the potential for better outcomes when combined with other drugs that could enhance its effect through synergy. Another potential therapeutic approach to consider is the use of intensive immunization with inactivated poliovirus vaccine. Since poliovirus and CVA1 belong to the same species, EV-C, there is a possibility of observing cross-neutralizing reactivity. Although evidence for such cross-protection is currently limited, this strategy might stimulate the production of antibodies that could partially neutralize CVA1. Further investigation into this strategy could offer valuable insights.

While CVA1 did not replicate in our gut organoid transwell model (data not shown), preliminary results from a co-culture model of paired human fetal intestinal epithelial cells and fibroblasts showed successful CVA1 replication. However, treatment with RDV did not significantly reduce viral replication, reflecting the patient's unfavorable response. Further experiments are needed to confirm these findings and evaluate the efficacy of the other compounds.

The clinically relevant concentration of 4 µM RDV, matching in vivo C_max_ values, proved nontoxic in the HIE model, highlighting its potential as a superior platform for drug toxicity evaluation compared to traditional cell lines. FLX showed no toxicity in either RD cells or the HIE model, consistent with its safety profile. However, given FLX's CNS-related side effects, like the malaise experienced by the patient, toxicity assessments should involve CNS-specific models, such as brain organoids. However, organotypic models, resembling only certain features of the respective organ, have limitations in evaluating systemic drug toxicity.

In summary, the vulnerability of immunocompromised patients to severe enterovirus infections necessitates urgent treatment. While RDV was ineffective in clearing CVA1 infection in this immunocompromised patient, it still holds potential, especially in combination with other drugs [57, 59]. Repurposing FDA-approved drugs offers advantages of established safety profiles and the possibility of rapid compassionate use.

## Notes

***Acknowledgments.*** The authors would like to acknowledge the laboratory of Medical Microbiology and Infection prevention at Amsterdam UMC, especially Marion Cornelissen, for viral typing; the HIS mouse facility (Amsterdam UMC) and K. Weijer, E. Siteur-van Rijnstra, and A. Voordouw for their help in providing fetal tissues for the study; and the molecular and enterovirus culture team at the National Institute of Public Health and the Environment for preparing the samples for full-length sequencing.

***Author contributions.*** Conceptualization: G. M., D. P., A. S., and K. C. W. Data curation: G. M., C. C., G. K., H. v. E., and N. J. Formal analysis: G. M. Funding acquisition: D. P., A. S., and K. C. W. Investigation: G. M., C. C., G. K., H. v. E., and N. J. Methodology: G. M., C. C., G. K., H. v. E., N. J., J. D. R., K. S. M. B., and J. C. Project administration: G. M., E. J. P., D. P., A. S., and K. C. W. Resources: G. M., C. C., G. K., H. v. E., N. J., J. D. R., K. S. M. B., J. C., and E. J. P. Validation and visualization: G. M. Writing–original draft: G. M., C. C., A. S., E. J. P., and K. C. W. Writing–review and editing: G. M., C. C., K. S. M. B., J. C., D. P., A. S., E. J. P., and K. C. W. All authors contributed to the article and approved the submitted version. All authors have read and agreed to the published version of the manuscript.

***Financial support.*** This work was supported by the European Union’s Horizon 2020 Research and Innovation Programme under the Marie Sklowdowska-Curie Grant Agreement OrganoVIR (grant number 812673 to K. S. M. B., D. P., and K. C. W.); the European Union’s Horizon 2020 Research and Innovation Programme under the grant agreement GUTVIBRATIONS (grant number 953201 to D. P., A. S., and K. C. W.); and the Public–Private Partnership Allowance (Focus-on-Virus) made available by Health Holland, Top Sector Life Sciences and Health, to the Amsterdam UMC, location Amsterdam Medical Center, to stimulate public–private partnerships.
